# Editorial: Recent innovations to inhibit scar formation for better skin regeneration

**DOI:** 10.3389/fsurg.2023.1325832

**Published:** 2023-11-02

**Authors:** Xiao-Ying Lin, Tao Zan, Xiao-Li Wu, Guang-Shuai Li, Wei-Qiang Tan

**Affiliations:** ^1^ Department of Plastic Surgery, Sir Run Run Shaw Hospital, Zhejiang University School of Medicine, Hangzhou, China; ^2^ Department of Plastic and Reconstructive Surgery, Shanghai Ninth People’s Hospital, Shanghai Jiao Tong University School of Medicine, Shanghai, China; ^3^ Department of Plastic and Reconstructive Surgery, The First Affiliated Hospital of Zhengzhou University, Zhengzhou, China

**Keywords:** wound healing, scar formation, scar treatment, mechanical force, negative pressure wound therapy, skin flap

**Editorial on the Research Topic** Recent innovations to inhibit scar formation for better skin regeneration

Scar formation after skin injuries and the challenges of managing keloids and chronic wounds have long been areas of concern in medicine and surgery. The socioeconomic burden of pathological scarring, both physical and psychological, highlights the urgency of continued research in this area. Therefore, the theme of this study is to inhibit scarring for better skin regeneration, and five articles are brought together to provide valuable insights into different aspects of wound healing, scar prevention, and keloid treatment by understanding the role of various cellular and mechanical force interactions in wound healing, as well as comparing different treatment strategies for wound repair in clinical and animal models.

In the article entitled *The Effects of Mechanical Force on Fibroblast Behavior in Cutaneous Injury*, Berry et al. delves into the cellular mechanisms behind pathologic scar formation. It explores the role of mechanical stress in wound healing and how it impacts fibroblast behavior. The review highlights key proteins and signaling pathways involved in mechanosensing, shedding light on potential therapeutic targets for scar reduction and improved wound healing. Additionally, it discusses clinical treatments aimed at reducing tension on wounds, offering hope for scarless healing in the future.

Wound management is a critical aspect of scar prevention, Tingting et al. compares two wound therapy techniques, standard negative pressure wound therapy (NPWT) and NPWT with instillation (NPWTi), in a porcine model. The study finds that NPWTi effectively reduces bacterial burden and virulence compared to standard NPWT, though it doesn't necessarily result in better histologic outcomes.

The next article also focuses on deep partial-thickness hand burns and the potential benefits of early rehabilitation combined with NPWT. Liu et al. shows that this combined approach significantly improves hand function, with better angle change and higher patient-reported outcomes compared to routine rehabilitation and NPWT alone. These researches contribute to our understanding of wound healing techniques and their potential benefits.

In the realm of reconstructive surgery, random skin flaps are commonly used but are prone to ischemia and necrosis. Therefore, it is critical to seek novel approaches to improve the clinical application of random flaps. The next article is an original study by Zhang et al. wherein the authors explore the use of limonin, a bioactive compound, in enhancing the survival of random skin flaps. The study demonstrates that limonin treatment reduces necrosis and edema, increases blood flow, promotes angiogenesis, and inhibits apoptosis and oxidative stress, all of which contribute to improved flap survival.

Finally, focusing on the treatment of keloids, Zhao et al. article presents a retrospective study on the use of internal mammary artery perforator propeller flaps in combination with radiotherapy for treating large chest keloids. The results demonstrate the effectiveness of this approach in reducing keloid recurrence and alleviating related symptoms, all while minimizing donor site damage and ensuring speedy postoperative recovery.

In summary, these five articles collectively advance our understanding of scar formation, wound healing, and the management of challenging clinical conditions such as keloids and deep burns. The insights presented in these papers hold promise for improved patient outcomes and better quality of life for those affected by pathologic scarring and complex wounds. As we continue to unravel the mysteries of wound healing, we look forward to further breakthroughs that will benefit patients and enhance the practice of medicine and surgery.

