# Corrigendum: Editorial: Recent innovations to inhibit scar formation for better skin regeneration

**DOI:** 10.3389/fsurg.2024.1351577

**Published:** 2024-01-10

**Authors:** Xiao-Ying Lin, Tao Zan, Xiao-Li Wu, Guang-Shuai Li, Wei-Qiang Tan

**Affiliations:** ^1^ Department of Plastic Surgery, Sir Run Run Shaw Hospital, Zhejiang University School of Medicine, Hangzhou, China; ^2^ Department of Plastic and Reconstructive Surgery, Shanghai Ninth People’s Hospital, Shanghai Jiao Tong University School of Medicine, Shanghai, China; ^3^ Department of Plastic and Reconstructive Surgery, The First Affiliated Hospital of Zhengzhou University, Zhengzhou, China

**Keywords:** wound healing, scar formation, scar treatment, mechanical force, negative pressure wound therapy, skin flap

A Corrigendum on Editorial: Recent innovations to inhibit scar formation for better skin regeneration By Lin X-Y, Zan T, Wu X-L, Li G-S and Tan W-Q. (2023). Front. Surg. 10:1325832. doi: 10.3389/fsurg.2023.1325832

Error in Author List

In the published article, there was an error in the author list, and author [Wei-Qiang Tan] was erroneously [ranked]. The corrected author list appears below.

Xiao-Ying Lin^1^, Tao Zan^2^, Xiao-Li Wu^2^, Guang-Shuai Li^3^, Wei-Qiang Tan^1^

The authors apologize for this error and state that this does not change the scientific conclusions of the article in any way. The original article has been updated.

